# Enhancing Clinical Practice in Adult Airway Suctioning: A Two-Cycle Audit at Aswan University Hospital

**DOI:** 10.7759/cureus.98372

**Published:** 2025-12-03

**Authors:** Alaa Altayeb Alsideeg Mohammed, Mustafa A AboAlella, Mohammed Ahmed Mohammed Dafaalla, Doha Mohamed Mousa Ali, Yasmin Mojahid Mohamed Jafar, Othman Yousuf Ibrahim Elhaj, Lujain Abdelhameed Bushra Ibrahim, Wejdan Mutwali Osman Mohammed, Abda Omer Osman Omer, Othman Hayder Ibrahim Saeed, Istabraq Rashad Ibrahim Adam, Rehab Rashad Ibrahim Adam, Shazly Bghdadi Ali Ahmed

**Affiliations:** 1 Medicine, Aswan University Hospital, Aswan, EGY; 2 Pathology and Laboratory Medicine, University of Bahri, Khartoum, SDN; 3 Pulmonary Medicine, Aswan University Hospital, Aswan, EGY

**Keywords:** adult airway suctioning, aswan, clincal audit, endotracheal suctioning, quality improvement projects

## Abstract

Background: Endotracheal suctioning (ETS) is a critical intervention for mechanically ventilated adult patients in intensive care units (ICUs), but improper technique can lead to significant complications. Adherence to established ETS guidelines is essential for patient safety and optimal outcomes. The aim of this study is to assess and improve healthcare professionals’ adherence to adult airway suctioning guidelines at Aswan University Hospital through a two-cycle clinical audit and targeted educational intervention.

Methods: A cross-sectional audit was conducted in the ICU, Emergency Department, and Short Stay Rooms at Aswan University Hospital. In the first cycle, 103 healthcare workers were evaluated for adherence to ETS protocols using a standardized questionnaire. Root causes of non-compliance were identified, followed by an educational intervention including didactic sessions, hands-on training, and protocol reinforcement. The second cycle involved 125 participants and reassessed adherence post-intervention.

Results: The educational intervention led to notable improvements in guideline adherence. Recognition of suctioning indications improved from 24 (23%) in the first cycle to 91 (72.8%) in the second. Performance of post-suctioning procedures rose from 21 (20%) to 100 (80%). Awareness of special considerations increased from 26 (25%) to 94 (75%). Moderate gains were also seen in suction duration, catheter selection, and complication awareness. However, documentation and equipment selection showed only modest improvement or minor decline, indicating the need for continued education.

Conclusion: Targeted education and protocol reinforcement significantly improved most aspects of airway suctioning practice among healthcare workers. Sustained improvements require ongoing training, regular audits, and a focus on practical skills, particularly in documentation and equipment selection.

## Introduction

Endotracheal suctioning (ETS) is an essential and frequently performed procedure in the management of adult patients requiring mechanical ventilation in intensive care units (ICUs) [1]. The primary objective of ETS is to maintain airway patency by removing secretions, which helps ensure adequate ventilation, oxygenation, and the prevention of complications such as atelectasis and ventilator-associated pneumonia (VAP) [2]. Despite its necessity, ETS is an invasive intervention that carries significant risks, including hypoxemia, cardiac arrhythmias, tracheal mucosal injury, and infection if not performed according to established guidelines [3].

International clinical practice guidelines, such as those from the American Association for Respiratory Care (AARC), emphasize evidence-based practices for ETS, including performing suctioning only when clinically indicated, using sterile techniques, selecting appropriate catheter size, and applying minimal negative pressure for no longer than 10-15 seconds per suction pass [4]. Strict adherence to these guidelines is vital to minimize adverse effects and improve patient outcomes [5].

However, studies have shown that adherence to ETS guidelines among critical care nurses and other healthcare professionals is often suboptimal, with variations in practice increasing the risk of complications for ICU patients [6]. Factors influencing adherence include the level of training, availability of protocols, and ongoing education. Therefore, regular assessment of adherence to ETS guidelines is crucial, particularly in resource-limited settings, to identify gaps in practice and implement targeted interventions for quality improvement [7].

At Aswan University Hospital, assessing adherence to ETS guidelines among adult ICU patients is essential for ensuring best practices, prioritizing patient safety, and reducing preventable complications. This study aims to assess the adherence of healthcare professionals to established ETS guidelines in the adult ICU setting at Aswan University Hospital, providing insights that can inform future training and policy development.

## Materials and methods

Study design and setting

This observational cross-sectional clinical audit was conducted at Aswan University Hospital, a major tertiary care center in Egypt serving a diverse urban and rural population. The audit evaluated healthcare professionals’ adherence to ETS guidelines across three clinical settings: the ICU, emergency department, and short stay rooms.

A total of 103 healthcare workers participated in the first cycle, and 125 in the second cycle after the educational intervention, yielding an overall sample size of 228 participants. All physicians actively working within the selected departments during the study period were included, ensuring a comprehensive representation of routine practice. Overall, the audit demonstrated notable improvement in adherence to ETS guidelines across most parameters following the intervention.

Questionnaire development and verification

A structured questionnaire (Appendix) was used for data collection in both cycles. The tool was developed based on nationally and internationally recognized airway suctioning guidelines to enhance reproducibility and standardization. To strengthen methodological rigor, the following were carried out:

1. The questionnaire was reviewed and verified by a senior consultant in critical care, ensuring content validity and alignment with accepted clinical standards.

2. It was administered by trained physicians who adhered to a uniform approach.

3. Responses were checked for completeness and accuracy prior to analysis.

Although the audit did not formally assess inter-rater reliability, the involvement of trained assessors and consultant verification helped reduce variability. Participant demographics and professional roles were not recorded, which may influence adherence patterns and was acknowledged as a limitation regarding external reproducibility.

Study phases and interventions

First Cycle (Pre-intervention State and Root Cause Analysis: February-March 2025)

Baseline data were collected using the verified structured questionnaire to assess compliance with established ETS protocols, including suctioning frequency, technique, equipment selection, and documentation quality. Analysis of baseline findings revealed inconsistent adherence, particularly in the use of closed suction systems and documentation practices. Complications such as transient hypoxemia and minor airway trauma were occasionally observed and were linked to technical lapses or prolonged suction duration.

A root cause analysis identified multiple contributing factors, including inconsistent protocol application, suboptimal equipment utilization, variability in suctioning technique, incomplete documentation, and significant gaps in staff knowledge and training.

Intervention (Education and Protocol Reinforcement: April 2025)

A targeted educational intervention was implemented to address identified deficiencies. The intervention included the following:

1. Didactic sessions covering current ETS guidelines.

2. Hands-on demonstrations to reinforce correct technique.

3. Distribution of visual aids and quick-reference guides across clinical areas.

Emphasis was placed on correct catheter sizing, appropriate suction pressure, aseptic technique, limiting suction duration to 10-15 seconds, and accurate documentation. Updated protocols were disseminated across all relevant units, and real-time compliance monitoring was introduced to encourage sustained improvement.

Second Cycle (Post-intervention Evaluation: May 2025)

Following implementation of the educational measures, adherence to airway suctioning guidelines was reassessed using the same consultant-verified questionnaire. Improvements were evaluated in terms of documentation completeness, consistency with best practices, and technique-related parameters. Staff feedback was obtained to identify persistent challenges, informing further protocol refinement and supplementary training as needed.

Data analysis

All 103 healthcare workers from the relevant units were included in the study, ensuring comprehensive representation across departments. A standardized questionnaire, meticulously designed in alignment with both national and international guidelines, was employed to assess adherence and practices. Data collection and analysis were conducted using Google Forms, allowing for efficient compilation and processing. Descriptive statistics were applied to quantify adherence rates and identify emerging trends, providing a clear snapshot of current practices and areas for potential improvement.

Standards for adult airway suctioning

Suctioning is indicated in patients presenting with audible or visible secretions, an ineffective cough, changes in ventilator parameters, abnormal auscultation findings, or self-reported discomfort. However, it is contraindicated in individuals with unstable cardiovascular or respiratory status, those at risk of bleeding, patients who have recently undergone airway, head, or neck surgery, individuals with elevated intracranial pressure, and in cases where nasopharyngeal or oropharyngeal suctioning is specifically contraindicated. Proper equipment selection and preparation are essential, including the use of a sterile, flexible, single-use catheter of appropriate size, a suction device capable of delivering up to 150 mmHg pressure, personal protective equipment (PPE), sterile water, and an oxygen source. The suctioning procedure involves performing hand hygiene, explaining the process to the patient, positioning them appropriately, pre-oxygenating, gently inserting the catheter, applying suction during withdrawal for no longer than 10-15 seconds, reassessing the patient, and documenting the intervention. Special considerations include using closed suction systems for ventilated patients, exercising caution with head-injured individuals, and modifying techniques and equipment for neonates and pediatric patients. Potential complications such as hypoxemia, trauma, infection, bronchospasm, and cardiac arrhythmias can be minimized through pre-oxygenation, gentle technique, sterile equipment use, and continuous monitoring. Documentation should include the time of suctioning, catheter size, suction pressure used, the amount and type of secretions removed, patient tolerance, and any observed complications.

Feedback and effectiveness assessment

The impact of the intervention was assessed by comparing adherence and documentation rates before and after the educational sessions. Structured feedback from healthcare providers was used to identify ongoing challenges and inform future improvements.

## Results

A total of 103 healthcare workers participated in the first audit cycle, and 125 in the second cycle following the educational intervention. Overall, there was a notable improvement in adherence to adult airway suctioning guidelines across most parameters.

The most significant improvement was observed in the identification of the most indicative sign that a patient may require suctioning, which increased from 24 (23%) to 91 (72.8%), a 49.8% absolute improvement. Marked gains were also seen in the performance of post-suctioning procedures, rising from 21 (20%) to 100 (80%), and in awareness of special considerations, which improved from 26 (25%) to 94 (75%). Knowledge of contraindications for nasopharyngeal suctioning also increased substantially, from 47 (46%) to 106 (84.8%), a 38.8% improvement.

Moderate improvements were observed in several other areas: appropriate approach to determining the need for suctioning improved from 67 (65%) to 97 (77.6%); knowledge of desirable catheter characteristics increased from 61 (59%) to 94 (75.2%); awareness of the recommended duration for each suction attempt rose from 40 (39%) to 93 (74.4%); and understanding of when suction should be applied during catheter insertion improved from 41 (40%) to 88 (70.4%). Knowledge regarding the correct depth for catheter insertion improved from 44 (43%) to 93 (74.4%) (a 31.4% improvement), while understanding of appropriate suction pressure rose from 54 (52.4%) to 99 (79.2%), a 26.8% increase.

Smaller gains were seen in training received on the suctioning procedure, increasing from 64 (62%) to 84 (67.2%); assessment before, during, and after suctioning, from 43 (42%) to 58 (46.4%); and documentation of the procedure, from 71 (69%) to 94 (75.2%). The ability to identify potential complications improved from 72 (70%) to 107 (85.6%), while knowledge of hypoxemia prevention measures showed a marginal rise from 56 (54%) to 69 (55.2%).

A few parameters demonstrated either minimal improvement or a decline. Knowledge of the general guideline for choosing the appropriate catheter size decreased slightly from 86 (83%) to 100 (80.1%), and understanding of potential problems associated with suctioning dropped from 71 (69%) to 73 (58.4%).

In summary, the implementation of targeted educational interventions resulted in measurable improvements in most aspects of airway suctioning practice among healthcare workers at Aswan University Hospital. However, certain areas, such as equipment selection and awareness of complications, require ongoing attention. Regular education and re-auditing are recommended to sustain and further enhance compliance with best practice guidelines. Table 1 summarizes the adherence differences between the first and second cycles, as well as the overall improvement.

**Table 1:** Airway Suctioning Audit Outcomes: Comparative Analysis of Knowledge and Practice Compliance Between First and Second Cycles

Parameter	First Cycle, N (%)	Second Cycle, N (%)	Percentage of Improvement
Training received on suctioning procedure	64 (62%)	84 (67.2%)	5.2
Indications for airway suctioning (appropriate approach to determining need)	67 (65%)	97 (77.6%)	12.6
Indications for airway suctioning (most indicative sign patient may require suctioning)	24 (23%)	91 (72.8%)	49.8
Contraindications for airway suctioning (potential contraindication for nasopharyngeal suctioning)	47 (46%)	106 (84.8%)	38.8
Equipment selection and preparation (general guideline for choosing appropriate catheter size)	86 (83%)	100 (80.1%)	-2.9
Equipment selection and preparation (desirable characteristics of a suction catheter)	61 (59%)	94 (75.2%)	16.2
Suctioning procedure (recommended duration for each suctioning attempt)	40 (39%)	93 (74.4%)	35.4
Suctioning procedure (when suction should be applied during catheter insertion)	41 (40%)	88 (70.4%)	30.4
Suctioning procedure (how deep should you insert the catheter during suctioning)	68 (66%)	103 (82.4%)	16.4
Suctioning procedure (amount of pressure to be used for suctioning)	20 (19%)	60 (48%)	29
Suctioning procedure (what to assess before, during, and after suctioning)	43 (42%)	58 (46.4%)	4.4
Procedures performed after the suctioning procedure	21 (20%)	100 (80%)	60
Special considerations	26 (25%)	94 (75%)	50
Potential complications and prevention (potential complications of airway suctioning)	72 (70%)	107 (85.6%)	15.6
Potential complications and prevention (what can help prevent hypoxemia during suctioning)	56 (54%)	69 (55.2%)	1.2
Potential complications and prevention (what problems can occur due to suctioning)	71 (69%)	73 (58.4%)	-10.6
Documentation (what should be documented after an airway suctioning procedure)	71 (69%)	94 (75.2%)	6.2

## Discussion

This audit at Aswan University Hospital revealed substantial improvements in adherence to ETS guidelines among ICU healthcare professionals following a targeted educational intervention. The results demonstrate that structured training and reinforcement of protocols can effectively address gaps in knowledge and practice, ultimately enhancing patient safety and care quality.

The most significant gains were observed in the identification of clinical indications for suctioning, performance of post-suctioning procedures, and awareness of special considerations. For instance, the ability to recognize the most indicative sign for suctioning improved by nearly 50%, while correct post-suctioning procedures rose from 20% to 80%. These findings are consistent with previous research showing that focused education and protocol reinforcement are associated with improved compliance and clinical outcomes in airway management [2,3]. Such improvements are critical, as inappropriate or delayed suctioning can increase the risk of complications such as hypoxemia, infection, and VAP [2-5].

Moderate improvements were seen in areas such as the appropriate approach to determining the need for suctioning, desirable catheter characteristics, and recommended suction duration. These results mirror international evidence that ongoing education and regular audits are necessary to maintain high standards in ICU care [4,5]. However, the relatively modest gains in staff training, assessment before/during/after suctioning, and documentation suggest that these areas may require more interactive or hands-on educational strategies, such as simulation-based training or bedside coaching [6-8].

Notably, some parameters showed minimal improvement or even a decline. Knowledge of the general guideline for choosing catheter size and understanding of potential problems related to suctioning decreased in the second cycle. This may reflect the complexity of these topics or the need for more frequent reinforcement and practical demonstration [1-7]. Additionally, the marginal improvement in hypoxemia prevention highlights the importance of not only didactic teaching but also real-time feedback and scenario-based learning [9,10].

Documentation practices improved only slightly, despite being an area of focus in the intervention. Comprehensive documentation is essential for quality assurance, continuity of care, and medico-legal protection [5,6]. The modest gains in this area indicate that documentation should be a continuous focus of both formal training and daily clinical supervision.

The audit also identified persistent challenges in equipment selection and complication awareness, suggesting the need for ongoing education, regular re-auditing, and perhaps the integration of checklists or reminders into daily practice. Similar findings have been reported in other resource-limited settings, where consistent adherence to evidence-based guidelines can be challenging due to staffing, workload, and resource constraints [7-9].

Limitations

This audit was conducted in a single tertiary center and relied on self-reported questionnaires, which may be subject to response bias. The study did not assess the long-term retention of knowledge or the direct impact on patient outcomes, such as rates of VAP or airway trauma.

Implications for practice

The findings support the integration of ongoing, structured education and regular audit cycles in ICU practice. Emphasizing practical skills, real-time feedback, and continuous monitoring is likely to yield further improvements in adherence to ETS guidelines and patient safety. Future interventions should consider more interactive and scenario-based approaches, especially for complex or nuanced aspects of airway management.

## Conclusions

The implementation of targeted educational interventions at Aswan University Hospital led to measurable improvements in most aspects of airway suctioning practice. Ongoing education, regular audits, and a focus on practical, hands-on training are essential to sustain these gains and address persistent gaps, particularly in equipment selection, complication management, and documentation.

## Appendices

**Table 2:** Airway Suctioning Procedure Questionnaire

Suctioning Questionnaire Table			
Section	Question Number	Question	Options
General Info	1	What is your current role?	a. Intern b. Nurse c. Resident
General Info	2	In which intensive care unit (ICU) do you work?	a. Cardiac b. Emergency c. General Surgery d. Isolation e. Internal Medicine f. Neurology g. Obstetrics and Gynecology h. PACU i. Pulmonary
General Info	3	How long have you worked in the ICU?	a. 1–3 years b. 3–5 years c. 1 year d. 5 years
General Info	4	Have you received training on the airway suctioning procedure?	a. No b. Yes
I. Indications for Suctioning	1	What is the BEST approach to determine suctioning need?	a. Assessment-based b. Audible secretions only c. Patient request d. Routine every 2 hours
I. Indications for Suctioning	2	Most indicative sign for suctioning?	a. Audible secretions (no distress) b. Ineffective cough c. Pharyngeal secretions d. Transient SpO₂ changes
II. Contraindications	1	Contraindication for nasopharyngeal suctioning?	a. Head trauma (↑ICP) b. Stable cardiovascular status c. Sputum sample needed d. Tracheostomy (no obstruction)
III. Equipment	1	Catheter size guideline?	a. 2× ET/trach tube diameter b. Largest available c. ½× ET/trach tube diameter d. Size irrelevant
III. Equipment	2	Desirable catheter characteristics?	a. Flexible + side holes b. Flexible + reusable c. Rigid + reusable d. Single-use + rigid
			Tolerance/temperature
IV. Procedure	1	Recommended suction duration per attempt?	a. 10–15 sec b. 20 sec c. 30 sec d. Until secretions clear
IV. Procedure	2	When to apply suction?	a. During insertion b. During withdrawal c. Continuously d. Doesn’t matter
IV. Procedure	3	Catheter insertion depth?	a. Deeper for all b. Just beyond ET tube c. Shallow (ET tip only) d. To carina
IV. Procedure	4	Suction pressure?	a. 150–200 mmHg b. 60–80 mmHg c. 70–150 mmHg d. 200 mmHg (case-dependent)
IV. Procedure	5	Pre/during/post-procedure assessments?	a. Not needed b. SpO₂ + ECG c. SpO₂ + ECG + BP d. SpO₂ only
IV. Procedure	6	Post-procedure actions?	a. All of the above b. Document details c. Monitor vitals d. None e. Oxygen session
V. Special Considerations	1	Preferred suction system for ventilated patients?	a. Any system b. Closed c. Contraindicated d. Open
VI. Complications	1	Potential complication?	a. Bradycardia b. Hypoxemia c. ↑SpO₂ d. ↓Lung function
VI. Complications	2	How to prevent hypoxemia?	a. Frequent suctioning b. Large catheter c. Pre-oxygenate d. Prolong suction
VI. Complications	3	Problems from suctioning?	a. All b. Hypotension/hypoxia c. Hypoxia/↑ICP/atelectasis d. Pneumonia/bleeding
VII. Documentation	1	What to document post-procedure?	a. Catheter size used and patient’s weight b. Amount and description of secretions obtained c. Time of the procedure and patient’s blood pressure d. Patient’s tolerance of the procedure and post-procedure temperature
