# Correction: Enhancing Clinical Practice in Adult Airway Suctioning: A Two-Cycle Audit at Aswan University Hospital

**DOI:** 10.7759/cureus.c462

**Published:** 2026-07-15

**Authors:** Alaa Altayeb Alsideeg Mohammed, Mustafa A AboAlella, Mohammed Ahmed Mohammed Dafaalla, Doha Mohamed Mousa Ali, Yasmin Mojahid Mohamed Jafar, Othman Yousuf Ibrahim Elhaj, Lujain Abdelhameed Bushra Ibrahim, Wejdan Mutwali Osman Mohammed, Abda Omer Osman Omer, Othman Hayder Ibrahim Saeed, Istabraq Rashad Ibrahim Adam, Rehab Rashad Ibrahim Adam, Shazly Bghdadi Ali Ahmed

**Affiliations:** 1 Medicine, Aswan University Hospital, Aswan, EGY; 2 Pathology and Laboratory Medicine, University of Bahri, Khartoum, SDN; 3 Pulmonary Medicine, Aswan University Hospital, Aswan, EGY

This article has been corrected to fix a typo related to the study dates: the study was conducted from February 2025 to May 2025 (as opposed to 2024). This has now been corrected.

